# The free energy of mechanically unstable phases

**DOI:** 10.1038/ncomms8559

**Published:** 2015-07-01

**Authors:** A. van de Walle, Q. Hong, S. Kadkhodaei, R. Sun

**Affiliations:** 1 Box D, School of Engineering, Brown University, Providence, Rhode Island 02912, USA; 2 Division of Chemistry and Chemical Engineering, California Institute of Technology, Pasadena, California 91125, USA

## Abstract

Phase diagrams provide ‘roadmaps' to the possible states of matter. Their determination traditionally rests on the assumption that all phases, even unstable ones, have well-defined free energies under all conditions. However, this assumption is commonly violated in condensed phases due to mechanical instabilities. This long-standing problem impedes thermodynamic database development, as pragmatic attempts at solving this problem involve delicate extrapolations that are highly nonunique and that lack an underlying theoretical justification. Here we propose an efficient computational solution to this problem that has a simple interpretation, both as a topological partitioning of atomic configuration space and as a minimally constrained physical system. Our natural scheme smoothly extends the free energy of stable phases, without relying on extrapolation, thus enabling a formal assessment of widely used extrapolation schemes.

## Abstract (web summary)

Phase diagrams are useful to describe the different states of matter, although for mechanically instable system the derivation of a free energy and thus of phase diagrams has been a challenge. Here, the authors propose a computational method to define the free energy of mechanically unstable phases.

## Graphical abstract

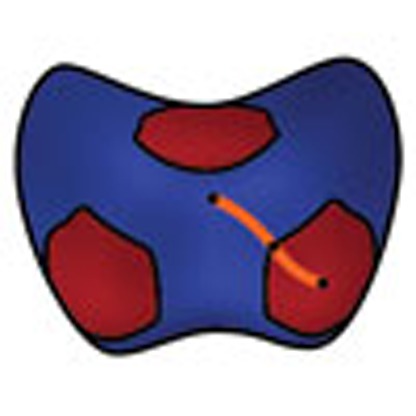

Phase diagrams play a central role in the physical sciences and computational methods have become a preferred route to obtain such thermodynamic information^1^,^2^,^3^,^4^,^5^. Widely used frameworks for computing phase diagrams (for example, computational thermodynamics^1^,^6^,^7^,^8^,^9^ and cluster expansion^10^,^11^,^12^,^13^,^14^ methods) rely on the assumption that all phases remain metastable with well-defined free energies under all conditions. Unfortunately, mechanical instabilities are common in solid-state systems^15^,^16^,^17^,^18^, making free energies undefined (other than by delicate extrapolation schemes). This problem has prompted a long search for reasonable and practical definitions of free energies for mechanically unstable phases^6^,^16^,^17^,^18^,^19^,^20^,^21^ and still hinders thermodynamic database development^21^. Here we propose an efficient computational solution with a natural interpretation, both as a topological partitioning of atomic configuration space and as a minimally constrained physical system. Our scheme smoothly extends the free energy of mechanically stable phases, without relying on extrapolation schemes. The results agree well with available computational and experimental estimates.

A common approach to handle mechanical instabilities is to merely assume that the functional form used to represent the data in a mechanically stable composition range will automatically provide a reasonable extrapolation into the unstable range. This ‘lattice stability' picture underlies the widely popular computational thermodynamics (also known as CALculation of PHase Diagram method, or CALPHAD) and cluster expansion (also called generalized Ising model) frameworks, but is not without well-documented problems^16^,^17^,^18^,^19^,^21^. Being intrinsically noisy, extrapolations from different directions in composition–temperature–pressure space may not agree. Without careful analysis, the extrapolated free energy of an unstable phase may inadvertently lie below the free energy of a truly stable phase. Conceptually, even defining the free energy of an unstable phase is difficult. Computational approaches are being increasingly used to provide independent corroboration to the CALPHAD assessments^22^ and to help address the problem of unstable phases^16^,^17^,^18^,^19^.

This report pursues this line of attack by proposing a general and efficient computational approach having a firmer theoretical basis that can be justified from three complementary points of view. First, it corresponds to a topological partitioning of phase space based on a simple curvature criterion. Second, it has the interpretation of stabilizing the system by constraining the minimum number degrees of freedom. Finally, the proposed definition yields free energies that vary smoothly as the system crosses the point of mechanical instability, a property that is verified both theoretically and through electronic structure calculations on actual alloy systems. The calculated free energies of unstable phases agree remarkably well with the ones obtained via existing extrapolation schemes, thus placing the latter on a stronger theoretical footing.

## Results

### Phase space partitioning

**Figure 1:**
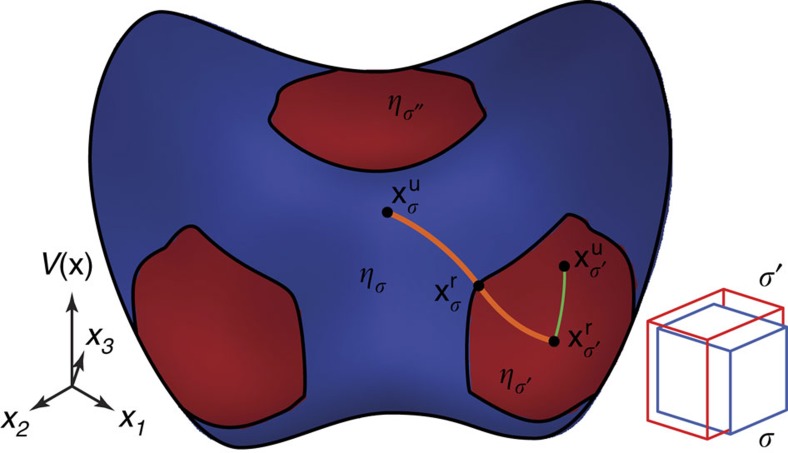
Partitioning of phase space into neighbourhoods. The potential energy hypersurface *V*(**x**) (as a function of the state **x** of the system) defines a natural partitioning of phase space into neighbourhoods *η*_*σ*_, based on the sign of *c*(**x**), the local minimum curvature of *V*(**x**) (blue: negative and red: positive). Each neighbourhood (stable or not) can be assigned a well-defined free energy by integration of the partition function over that neighbourhood. In this example, the point 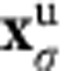 corresponds to fcc W while the point 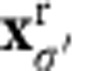 corresponds to bcc W (there are three symmetrically equivalent basins corresponding to bcc W) and the path joining them is the well-known Bain path. Also shown are paths of steepest descent from given ‘unrelaxed' positions (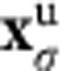, 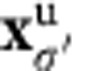) towards corresponding minima (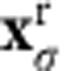, 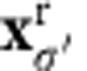) within the corresponding neighbourhoods (*η*_*σ*_, 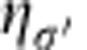). For the mechanically stable phase, 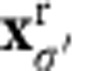 lies at a local minimum while for the mechanically unstable phase, 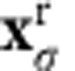 lies at an inflection point along a path of steepest descent.

From a topological point of view, phase space can be naturally partitioned into configurations *σ* based on the curvature of the system's energy hypersurface (Fig. 1). In a system of *N* atoms, let **x** denote the 3*N* vector of all atomic positions, let *V*(**x**) denote the potential energy of the system in that state and let *c*(**x**) be the minimum curvature at **x**, that is, the minimum eigenvalue of of the Hessian (the matrix of second derivatives). Hence, *c*(**x**) >0 and *c*(**x**) ≤0 correspond to mechanically stable and unstable regions, respectively. Within the lattice stability picture, a given lattice L is associated with many possible ways of assigning the atoms to the lattice sites. For each assignment *σ*, we denote the ideal (or ‘unrelaxed') positions of the atoms by 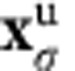. (For each *σ*, 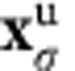 is a 3*N* vector.) In the neighbourhood of each 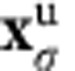, a small fraction of phase space around 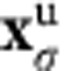, denoted *η*_*σ*_, is also associated with the configuration *σ*, to account for static and/or dynamic displacements of the atoms around their ideal lattice positions. The free energy obtained from the classical configurational partition function associated with lattice L is then given by a 3*N*-dimensional integral in a familiar ‘coarse-grained' form^11^:


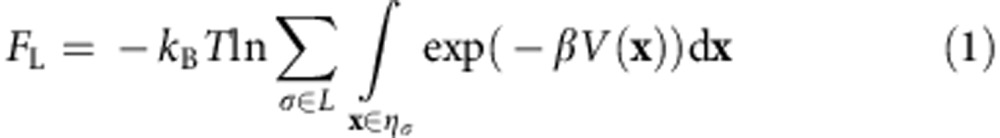


where *β*=1/(*k*_B_*T*), *T* is temperature and *k*_B_ is Boltzmann's constant. In our scheme, the neighbourhood *η*_*σ*_ is the largest connected set containing 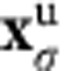 over which the minimum curvature *c*(**x**) does not change sign. Within the set *η*_*σ*_, we also define 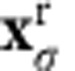 as the location of the minimum of *V*(**x**) within *η*_*σ*_ (that is, the ‘relaxed' positions) and 
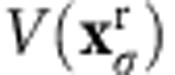
 is the energy of the system in configuration *σ* at 0 K. (Supplementary Note 1 provides more details regarding these definitions.)

When a configuration 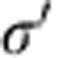 corresponds to a standard mechanically stable phase, this definition agrees with the usual notion of a local potential well: if one were to ‘pull' on the atoms with an increasing force, the system would break free of a given local minimum precisely at the point where a local instability develops (that is, when the minimum eigenvalue of the Hessian vanishes). For a mechanically stable phase, relaxation along a path of steepest descent from 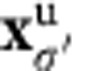 towards 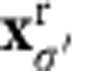 would always face a positive curvature along the path. In this case, the integral in equation (1) can easily be evaluated (for example, within a harmonic approximation around 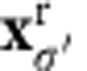 via a standard lattice dynamics calculation).

When a configuration *σ* is associated with a mechanically unstable phase, the point of minimum energy 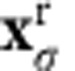 in *η*_*σ*_ is necessarily at its boundary (for otherwise the Hessian would have had to be positive definite at an interior local minimum). Finding this minimum generally involves solving a nonlinear constrained optimization problem. Checking if a given point belongs to *η*_*σ*_ at each step of the optimization can be accomplished with the dimer method^23^, which efficiently determines the smallest eigenvalue of the Hessian.

Fortunately, in the rather common case where the instability appears tangentially to a minimum energy path joining 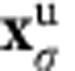 and a neighbouring local minimum 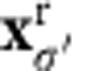 (Fig. 1), the point 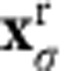 of minimum energy in *η*_*σ*_ can be easily determined as the inflection point of a path determined by the nudged elastic band method^24^. The cases where the onset of instability does not occur parallel to the path can be readily detected by performing a standard phonon analysis around the inflection point. If no other unstable mode is detected in this fashion, one can be confident that the inflection point is the appropriate point 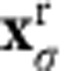 (since we have thus already identified the point of instability onset). This is the situation we encountered in the cases considered in this study. Note that there may be multiple possible distinct paths, in which case the one leading to the minimum *V*(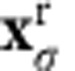) determines 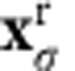. Different possible paths can be explored by considering various specific end points of the nudged elastic band path (for example, different nearby minima). This ‘path of steepest descent' picture bears some resemblance with Vineyard's transition state theory^25^, with the important distinction that an inflection point (rather than a saddle point) plays a key role in separating the different regions.

### Minimally constrained system

This idea of identifying the onset of instability also leads to a second characterization of the approach as using a minimally constrained system. At 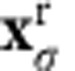 (and at any other point of the boundary of *η*_*σ*_), the system is mechanically stable along all but one direction. (Due to point group symmetry, a few directions may simultaneously become unstable at the same point.) Since in the thermodynamic limit (*N*→∞), the contribution of a single (or a finite number of) mode is negligible, a harmonic expansion around 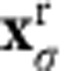 is still possible and a standard lattice dynamics calculation can be performed (unlike the method proposed in ref. 18). (Other methods may be used to provide a higher accuracy or to handle the presence of multiple nearby minima, as detailed in Supplementary Note 1.) The thermodynamic limit thus justifies merely neglecting the few unstable modes and the system's free energy can be defined by imposing a linear constraint that freezes the few unstable modes at 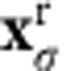. Our scheme can thus be viewed as constructing a ‘minimally constrained' physical system. Further advantages of this interpretation are discussed in Supplementary Note 2.

In this scheme, one may be concerned that a single unstable phonon mode can be surrounded (on the same phonon branch) by infinitely many stable, but very soft, modes that may still lead to a divergence of the free energy. However, as observed in ref. 19, the free energy contribution of a phonon branch with a single point where frequency vanishes involves the integral of a singularity that is only logarithmic and thus yields a finite contribution.

Our partitioning scheme may appear somewhat oversimplified in that it only checks for the mere presence of unstable mode(s) instead of distinguishing regions that exhibit a different number of unstable modes. However, such distinction brings little more, as one has to realize that, in an extended solid, when a phonon branch touches the zero frequency axis only a small finite number of unstable modes appear. Beyond that, when a phonon branch crosses the zero frequency axis, one then has an infinite number of unstable modes. So the possible numbers of unstable modes are only 0, *m*, infinity, where *m* is a small number (that does not scale with system size). The value *m* is only reached at the boundaries of the regions *η*_*σ*_ (where 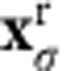 is located for mechanically unstable phases), while the values 0 or infinity are reached inside mechanically stable or unstable regions, respectively.

### Smooth extrapolation property

A third way to motivate the proposed approach is to study the behaviour of the system when some external variable *α* (for example, chemical composition or pressure) is continuously varied until the system crosses the point of mechanical stability at *α*=*α*_0_. To this effect, we consider a parameter-dependent potential *V*(**x**,*α*). As shown in Supplementary Note 3, the (constrained) Helmholtz free energy of the system at positive temperature in neighbourhood *η*_*σ*_ is a smooth function of *α* because it is given by the integral 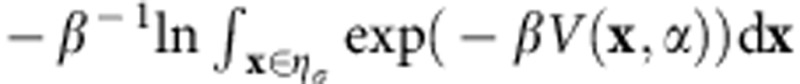 in which both *V*(**x**,*α*) and *η*_*σ*_ vary smoothly in *α*, except in rare singular cases.

**Figure 2:**
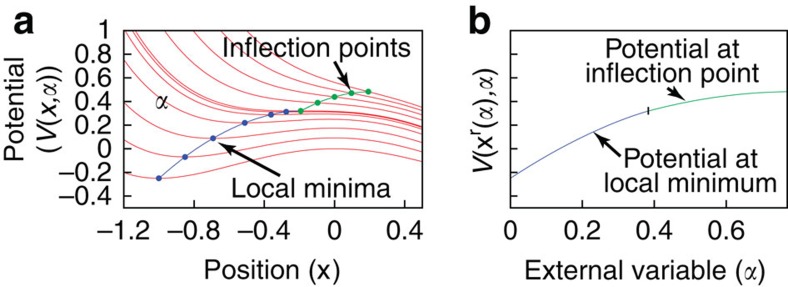
Demonstration of the smooth extrapolation property of the proposed approach. Example of one-dimensional potential (red curves) (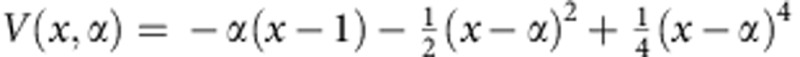) varying with the value of some external variable *α* (such as composition). (**a**) The energy (at 0 K) is given by the local minimum (blue) if it exists, and by the inflection point (green) when instability occurs. (**b**) The energy thus defined is a smooth function of *α*, even at the onset of instability (marked by a vertical dash).

The limit of zero temperature (where the free energy reduces to the energy) is useful (for example, in the context of cluster expansions) and provides the most stringent test of smoothness property (since the free energy integral reduces to the evaluation of the potential *V*(**x**) at a single point 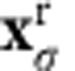, eliminating the automatic smoothing effect of the integral). To study this, we track the location of 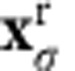 as a function of *α* (which we denote **x**^r^(*α*)) and as well as the corresponding value of the potential *V*(**x**^r^(*α*),*α*). Under our scheme, for *α*<*α*_0_, **x**^r^(*α*) is at a local minimum, while for *α*>*α*_0_, **x**^r^(*α*) tracks an inflection point. The disappearance of the local minimum at *α*=*α*_0_ signals the point beyond which there no longer exists a well-defined structural energy (at 0 K) in the traditional sense. Our approach then provides a way to assign an energy via the inflection point. We can assume *x* to be one-dimensional without significant loss in generality by considering, once again, the potential along a path joining 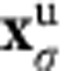 and 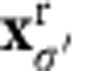. We show in Supplementary Note 4 that *V*(**x**^r^(*α*),*α*) is a continuously differentiable function of *α* at *α*=*α*_0_, so our scheme provides a smooth extension of the usual notion of local energy minimum. The fact that the minimum and the inflection point merge just indicates that the two notions agree in the limit case where they are both applicable, which is an arguably desirable property. This property is illustrated in Fig. 2 and follows the fact that, when a local minimum disappears, it does so by joining a nearby inflection point, which survives in the unstable regime (as further illustrated in Supplementary Fig. 1). This smooth extrapolation property is extremely convenient in the context of phase diagram calculations, because a series approximation (for example, by orthogonal polynomials) of the system's energy that covers both mechanically stable and unstable regions converges faster if the junction between the two regions is smooth^26^ (although the junction is not sufficiently smooth to allow for a Taylor expansion across the junction). This holds for both the cluster expansion and the polynomial expansions used in CALPHAD modelling.

### Application to alloy systems

We now demonstrate, through electronic structure calculations, that the above formal considerations actually lead to practically useful definitions of the free energy of mechanically unstable phases that (i) exhibit excellent smoothness properties and (ii) agree with available estimates. As a benchmark, we chose the Ir–Re–W alloy system, because it is relevant to the design of substitute alloys for Re in high-temperature applications^27^ and because it combines elements that each favours a different lattice: Ir is face-centered cubic (fcc), Re is hexagonal close-packed (hcp) and W is body-centered cubic (bcc). We compute the formation energies of ideal solid solutions as a function of composition, which simultaneously tests the suitability of our scheme for CALPHAD and cluster expansion modelling.

Our approach can be implemented by finding a path of steepest descent connecting the unrelaxed ideal lattice structure to the fully relaxed structure in one of two ways: (i) by using the nudged elastic band method, generalized to allow for cell shape variations^28^ or (ii) by using ‘damped' dynamics in which the atoms are repeatedly displaced in the direction of the force they experience by a fixed distance. The first option is useful if the relaxed structure has a lower symmetry than the unrelaxed one, while the second is computationally more efficient. In accordance with our definition of *η*_*σ*_ and 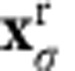, we determine the energy of the inflection point along that path, if it exists, and the energy of the fully relaxed structure, if it does not, thus suggesting the name ‘inflection-detection' method. A standard phonon analysis is then performed about the point of expansion identified in the previous step. If the number of unstable modes is negligible (that is, does not scale linearly with the density of the *k*-point mesh), the free energy can be obtained from the phonon density of states of the stable modes in the usual way^11^. If the number of unstable modes is not negligible then a more expensive search for the approriate expansion point must be performed using the dimer method to identify points of zero minimum local curvature, from which the minimum energy point must be determined.

When searching for the nearest local minimum, one needs to allow for possible symmetry breaking (for instance, in the case of fcc W, distortions along the Bain path must be allowed so that the nearby minimum is actually bcc W). To obtain phonon entropy contributions, we simply use a harmonic approximation about 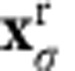 and a standard lattice dynamics analysis (the single unstable mode actually has null probability of falling exactly on one of the sampled *k*-points, so it requires no special treatment in our approach).

**Figure 3:**
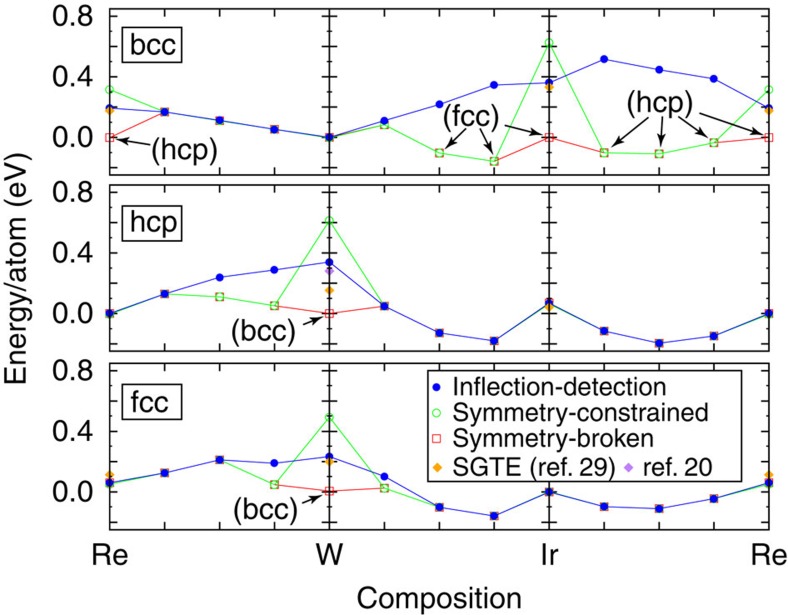
Formation energies of ideal solid solutions in all binary subsystems of the Ir–Re–W alloy system, obtained via various methods. Over mechanically stable regions, all three calculation methods considered agree. However, the proposed inflection-detection method provides the smoothest extrapolation behaviour into mechanically unstable regions, unlike formation energies obtained with symmetry-constrained relaxations (in which the structure's space group is not allowed to change during relaxations). At the same time, the inflection-detection method avoids full relaxation towards nearby mechanically stable phases (marked in parentheses), unlike relaxations allowing for symmetry breaking (where the relaxed structures are allowed to have a lower symmetry than the corresponding unrelaxed ones). The resulting inflection-detection predictions for pure elements agree remarkably well with the widely used SGTE values^29^ and more recent estimates^20^. (Formation energies are reported relative to each element's most stable crystal structure.)

In Fig. 3, mechanically unstable composition ranges are readily seen where there is no overlap between the results obtained with inflection-detection and those obtained with full relaxations allowing for symmetry breaking. Formation energies obtained by relaxing the atomic geometry under symmetry constraints (such that the unrelaxed and relaxed geometries have the same space group) are seen to have poor extrapolation behaviour, because the symmetry changes discontinuously with composition (solid solutions have a low local symmetry, while pure end members have a high symmetry). Formation energies obtained under relaxations allowing for symmetry breaking exhibit a somewhat smoother behaviour, but unfortunately allow the system to move away from the intended mechanically unstable phase into the nearby mechanically stable phase (marked in parentheses). The proposed ‘inflection-detection' method clearly yields the most natural extrapolation behaviour while at the same time avoiding full relaxation towards nearby mechanically stable phases. In addition, the inflection-detection predictions for pure elements agree remarkably well with the widely used Scientific Group Thermodata Europe (SGTE) values^29^, thus placing them on a stronger conceptual footing. This agreement is most likely the consequence of the smooth extrapolation property of the inflection-detection scheme. While agreement appears less good for the W hcp–bcc difference, this particular experimental estimate is based on limited data. For instance, the entropy difference between these phases is assumed to be zero in the SGTE data and an estimate which does not rely on this assumption^20^ agrees significantly better.

**Figure 4:**
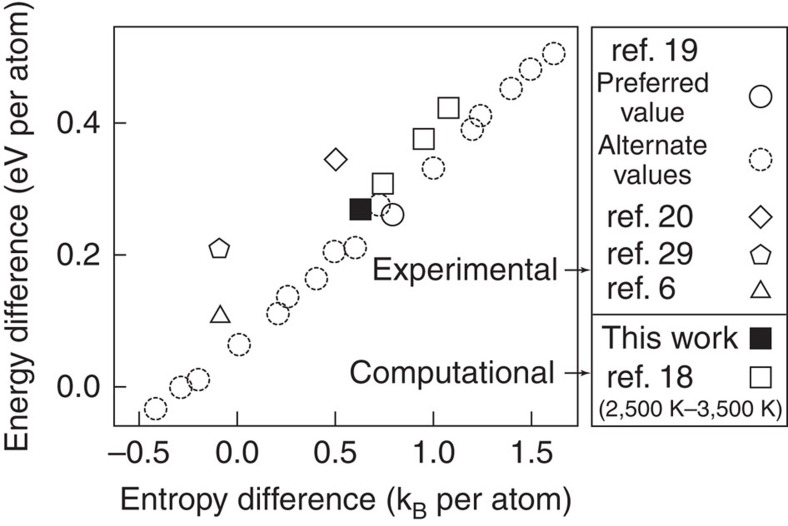
Proposed values of the energy and entropy differences between mechanically unstable fcc W and stable bcc W. The scatter in the available experimental estimates illustrates the inherent uncertainty of existing extrapolation schemes. Nevertheless, the inflection-detection method does corroborate the consensus value that seems to emerge from mutually consistent experimental^19^,^20^ and computational^18^ estimates.

It is instructive to further look into the case of fcc W, because it has received considerable attention. Figure 4 shows that the inflection-detection method yields values of the fcc–bcc energy and entropy difference in very good agreement with recent computations^18^. There is considerable spread in the specific CALPHAD estimates (because many postulated unstable free energies yield the same observable phase boundaries), but there is a clear ‘band' of energy–entropy combinations that are generally considered consistent with observed phase boundaries and our estimate falls in the middle of this band. Supplementary Note 5 provides additional details regarding the construction of this figure and Supplementary Fig. 2 displays the same data in an alternative representation (free energy difference as a function of temperature).

## Discussion

Caution is advised before blindly applying the proposed method to all phases exhibiting some form of mechanical instabilities. If a mechanically unstable state *σ* can potentially relax to many multiple nearby minima, a local analysis of the free energy based on an expansion about the inflection points may need to be supplemented by an analysis of the degeneracy of the paths (which will introduce an additional entropy contribution). However, if the number of paths does not increase exponentially with system size, the corresponding entropy contribution is negligible in the thermodynamic limit. This is the case in the examples considered in this report, but would not be the case, for instance, for bcc titanium^30^. The case of bcc Ti is an example of a high-symmetry crystal structure that is mechanically unstable based on a harmonic phonon analysis but that is nevertheless ‘dynamically stabilized' because the structure hops between local minima away from the high-symmetry point where mechanically instability occurs. (This can even take place in such a way that the average position of the atoms is at the high-symmetry configuration.) In such cases, the general formalism of partitioning phase space based on curvature still holds, but the dynamically stabilized phase corresponds to the collections of local mechanically stable potential wells located away from the high-symmetry point, not the mechanically unstable region containing the high-symmetry point. These many different configurations *σ* must be accounted for to yield the correct free energy and hence a harmonic expansion about a single local minimum is insufficient. The multiple local minima can be conveniently handled via cluster expansion techniques^10^,^11^,^12^,^13^,^14^. This situation is rather different from the type of mechanical instability considered in this paper, because bcc Ti actually exists in nature while the phases we consider here (such as fcc W) do not (making the definition of their free energy more challenging).

In summary, we propose a very simple and efficient method to define the free energy of mechanically unstable phases that is grounded in formal statistical mechanics and that exhibits the desirable smooth extrapolation behaviour into unstable regions that makes a direct connection with existing extrapolation schemes. Extensive testing on a particularly challenging ternary system exhibiting all three common lattices (fcc, bcc and hcp) confirms the predicted smooth behaviour and shows very good agreement with available experimental and computational estimates. The proposed method has been implemented within the Alloy Theoretic Automated Toolkit (ATAT)^31^,^32^,^33^ as the ‘robustrelax' command.

As such, the method offers promising avenue to solve the mechanical stability problem in the computational thermodynamics and cluster expansion frameworks that should benefit the large community of researchers in industry and academia that depends on phase diagrams to solve materials design problems in the automotive, aerospace, chemical process and electronic industries as well as in the general fields of materials science, solid-state physics, chemistry and planetary sciences.

## Methods

### Electronic structure

Electronic structure calculations were performed with the Vienna Ab initio Simulation Package (VASP) code implementing the projector augmented wave method^34^. The PBE (Perdew, Burke and Ernzerhof) functional^35^ was used, with a plane wave kinetic energy cutoff of 250 eV. The *k*-point mesh was automatically determined, as described in ref. 31, to guarantee at least 4,000 *k*-points per reciprocal atom. For ionic relaxations and force calculations (forces converged to 10^−2^ eV Å^−1^), Fermi smearing of 0.1 eV was used with the Methfessel–Paxton scheme^36^ of order 1. For total energy calculation on prerelaxed geometries, the tetrahedron method with Blöchl corrections^37^ was used. Phonon calculations were performed with the supercell method using the fitfc code^33^ including up to third nearest neighbour force constants in a 64 atom supercell with 0.2 Å displacements.

### Solid solution model

Solid solutions were modelled using Special Quasirandom Structure (SQS)^38^, generated by the mcsqs code^39^. These SQS contain 32–48 atoms and ensure that at least the third nearest neighbour pair correlations match those of the disordered state and that all three-body correlations with a diameter below the third nearest neighbour pairs do not deviate by >1/8 from their disordered state values.

### Steepest descent path determination

Nudged elastic band method calculations were performed with six images connecting an ideal unrelaxed structure to its fully relaxed counterpart. For systems with periodic boundary conditions (as considered here), the ‘unrelaxed' structure is obtained by freezing all ionic degrees of freedom as well as all cell shape parameters, except for the volume, which is allowed to equilibrate (this eliminates numerical difficulties associated with very large and unphysical energy changes). To determine the fully relaxed structure, symmetry breaking was necessary for the high-symmetry pure elements. Symmetry breaking was along the Bain path for fcc–bcc instabilities and along the Burger path for bcc–hcp instabilities. For the SQS, symmetry breaking was unnecessary. When six images were found to be insufficient to resolve the energy landscape (this issue was concentrated in the bcc SQS structures), a more efficient steepest descent algorithm was used to decompose the path into 10–80 images. In all cases, piecewise cubic polynomial interpolation was used to locate the inflection point accurately.

## Additional information

**How to cite this article:** van de Walle, A. *et al.* The free energy of mechanically unstable phases. *Nat. Commun.* 6:7559 doi: 10.1038/ncomms8559 (2015).

## Supplementary Material

Supplementary Information Supplementary Figures 1-2, Supplementary Notes 1-5 and Supplementary References
