# Supplementary material for: The free energy of mechanically unstable phases
Source: Nat Commun. 2015 Jul 1;6:7559. doi: 10.1038/ncomms8559 (PMC4506496; doi:10.1038/ncomms8559)
Supplement: Supplementary Information — Supplementary Figures 1-2, Supplementary Notes 1-5 and Supplementary References [file ncomms8559-s1.pdf]

Supplementary Figure 1

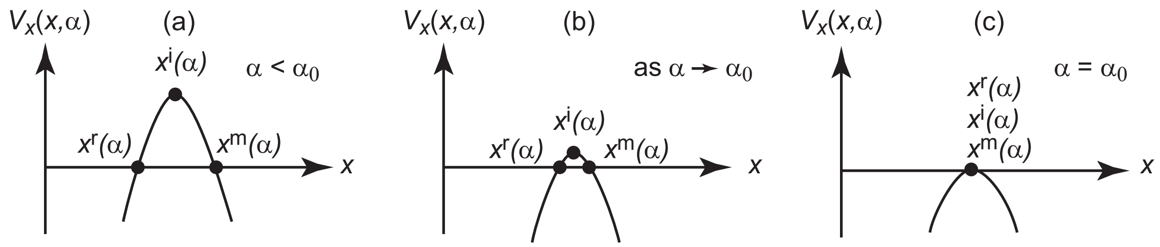

Supplementary Figure 1: **Behavior of the potential energy in the viscinity of the onset of mechanical instability.** The first derivative of the potential energy of the system $V_x(x, \alpha)$ as a function of the system's state x is represented, with panels (a),(b) and (c) showing the effect of varying the value of some external parameter α (such as composition or pressure). This illustrates that the disapperance of a local minimum $x^r(\alpha)$ (in panel (c)) must be accompanied by merging of an inflection point $x^i(\alpha)$, a local minimum $x^r(\alpha)$ and a local maximum $x^m(\alpha)$ at $\alpha = \alpha_0$.

Supplementary Figure 2

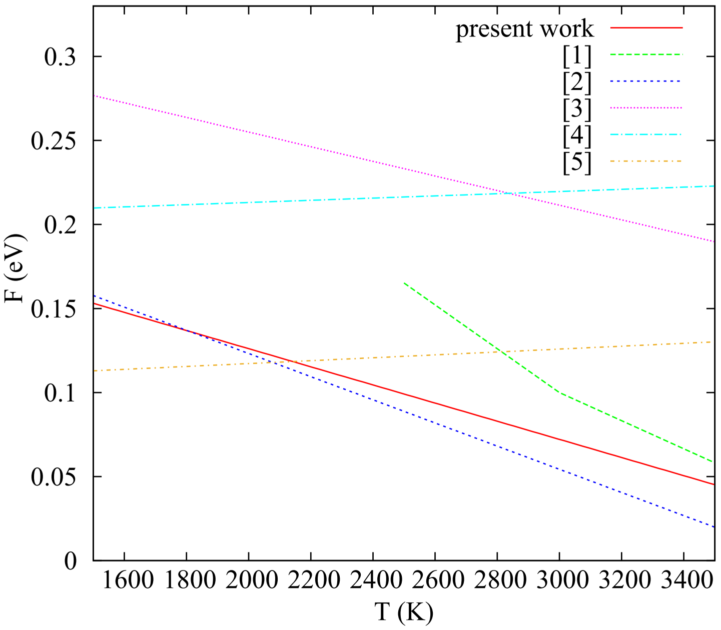

Supplementary Figure 2: **Calculated free energy difference between fcc and bcc W as a function of temperature.** Existing results [1–5] also shown for comparison.

Supplementary Note 1: Additional details regarding the proposed partitioning of phase space

1. If a periodic system is considered, then the variable $\mathbf{x}$ also includes the cell parameters of the unit cell enclosing one period of the whole system. For unit consistency, we define these cell degrees of freedom as strains relative to a reference state, scaled by average atomic volume in that reference state to the power 1/3 (in accordance with [6]).

2. For conciseness, we omit the inconsequential kinetic energy term in the partition function in Equation (1). Also, particle indistinguishability is implicitly accounted for in the summation over σ.

3. The classical partition function in Equation (1) is used for simplicity of exposition, the corresponding quantum mechanical expression (including kinetic energy contributions) is [7]:
$$F_L = -k_{\mathrm{B}}T \ln \sum_{\sigma\in L} \sum_i \langle\psi_{\sigma,i}| \exp(-\beta\mathbf{H})|\psi_{\sigma,i}\rangle$$
where $\mathbf{H}$ is the system's Hamiltonian and $\psi_{\sigma,i}$ is a complete orthogonal basis of functions supported on η_σ.

4. The neighborhoods η_σ are defined by connected sets where either $c(\mathbf{x}) > 0$ or $c(\mathbf{x}) \leq 0$. Note that we purposely group the value 0 with the negative values of $c(\mathbf{x})$, because the minimum energy occurs at the boundary in that case and it is thus logical to include the boundary in the set of possible solutions.

5. It may happen that, along a **given** path joining a mechanically stable and a mechanically unstable state, the onset of instability does not occur at the inflection point and is instead driven by the apparition of unstable modes perpendicular to the given path [8–10]. When this happens, it usually means that the given path is along a ridge of the potential surface, so there exists another, lower energy, path in which the mechanical instability does occur at the inflection point and that would be the relevant expansion point for a harmonic treatment. The rare cases where the onset of instability does not occur along the path (and no lower energy path exist) can be readily detected by performing a standard phonon analysis around the inflection point. If the number of unstable modes does not scale with the number of k-points sampled, then the inflection point found is a suitable harmonic expansion point: these modes are negligible in the thermodynamic limit, and thus require no special treatment (they can be neglected in the partition function calculation). If the number of unstable modes does constitute a fixed fraction of all modes, then the instability did appear perpendicular to the path of steepest descent (at another point of the path). One then has to revert to a more expensive algorithm (that checks the curvature along any directions, say with the dimer method or via repeated lattice dynamics analysis) to identify the point of minimum energy within the unstable region.

6. For increased accuracy, the harmonic approximation about $\mathbf{x}_\sigma^{\mathrm{r}}$ can be replaced by either, higher-order expansions, a quasiharmonic treatment or molecular dynamics simulations. In each case, the simulations must be constrained to a hyperplane intersecting some point $\mathbf{x}_\sigma^*$ and normal to the gradient of the potential at $\mathbf{x}_\sigma^*$. For higher-order expansions, $\mathbf{x}_\sigma^* = \mathbf{x}_\sigma^{\mathrm{r}}$, while for quasiharmonic and molecular dynamics, the point $\mathbf{x}_\sigma^*$ is somewhere along a path of steepest descent located between $\mathbf{x}_\sigma^{\mathrm{u}}$ and $\mathbf{x}_\sigma^{\mathrm{r}}$ and is chosen so as to minimize the (constrained) free energy, in analogy with the usual quasiharmonic approximation or cell-constrained molecular dynamics.

7. If the geometry of the neighborhood η_σ is complex, a path of steepest descent joining $\mathbf{x}_\sigma^{\mathrm{u}}$ to $\mathbf{x}_\sigma^{\mathrm{r}}$ may temporarily leave η_σ. In such a case, it may be necesary to try alternative starting points (instead of $\mathbf{x}_\sigma^{\mathrm{u}}$) to ascertain which inflection point is the appropriate one. This situation is akin to the well-known difficulty in finding the global (instead of a local) minimum energy in the space of crystal structures.

8. Within the harmonic approximation, if one wishes to account for the contribution of the neglected unstable mode perpendicular to the boundary of η_σ at $\mathbf{x}_\sigma^{\mathrm{r}}$, this can easily be done since, a quadratic expansion about an inflection point is actually linear, so the partition function along that dimension, within η_σ, just reduces to the convergent integral of an exponential function, truncated at the inflection point.

9. Although our curvature criterion provides a convenient method to divide phase space, it can also be combined with other approaches for computational convenience or efficiency. For instance, distinct regions under our scheme can be combined and labelled as a single configuration if they share very similar structural features (for instance if the unstable region surrounding the initial position $\mathbf{x}_\sigma^{\mathrm{u}}$ is very small and one thus wishes to merge it with a nearby stable region). This could be achieved by assigning the same configuration σ to any local minimum that is located within a given small distance d from a given initial unrelaxed position $\mathbf{x}_\sigma^{\mathrm{u}}$. At the other end of the spectrum, one unstable region could englobe mutiple different "pockets" corresponding to rather distinct geometries connected by high-energy corridors. In this case, it may be useful to separate these regions and assign them different configurations σ. As long as the boundaries created between the regions are in the high-energy corridors, the corresponding free energies should not be very sensitive to the precise location of the boundaries, because the local minima $\mathbf{x}_\sigma^{\mathrm{r}}$ corresponding to each region η_σ will be located at other points (where the minimum curvature vanishes) of much lower energy.

Supplementary Note 2: Cell-constrained molecular dynamics method

In this section, we note some unexpected features of the cell-constrained ab initio molecular dynamics method proposed in [1] to calculate constrained free energy difference (with a focus on fcc and bcc W). In essence, that method freezes the system's unstable modes and considers how the stable modes in the fcc structure are modified as the system is smoothly transformed into a bcc structure, resulting in a well-defined constrained free energy difference

$(F_c^{fcc} - F_c^{bcc})$ between fcc and bcc W. However, Ozolins cautions against the interpretation of $(F_c^{fcc} - F_c^{bcc})$ as a genuine free energy difference $(F^{fcc} - F^{bcc})$.

Here we expand a little more on the nature of the problems encountered when using $(F_c^{fcc} - F_c^{bcc})$ as if it were $(F^{fcc} - F^{bcc})$. If one were to use $(F_c^{fcc} - F_c^{bcc})$ to build a thermodynamic function database for W, one has to be aware of the following unintended implication: The implied value for "unconstrained" fcc W free energy F^{fcc} effectively replaces the missing contributions from the fcc unstable phonon modes by the corresponding contributions from the stable bcc modes.

This can be seen as follows: In a CALPHAD framework, the fcc free energy F^{fcc} would be expressed in term of the bcc free energy F^{bcc} and the constrained free energy difference $(F_c^{fcc} - F_c^{bcc})$ as:

$$F^{fcc} = (F_c^{fcc} - F_c^{bcc}) + F^{bcc}.$$

Consider a harmonic system in the high-temperature (or classical) limit for simplicity of exposition (since the proposed method should be valid at least for a harmonic system at any temperature). The contribution of each phonon mode i with frequency ν_i then has the form $-k_\mathrm{B}T\ln(h\nu_i/k_\mathrm{B}T)$. Letting $\mathcal{S}$ denote the set of modes that are stable in fcc and assuming the vibrational modes of each structure can be mapped one-to-one (breaking degeneracies if needed), we have

$$\begin{aligned} F^{fcc} &= -\sum_{i\in\mathcal{S}} k_\mathrm{B}T\ln(h\nu_i^{fcc}/k_\mathrm{B}T) + \sum_{i\in\mathcal{S}} k_\mathrm{B}T\ln(h\nu_i^{bcc}/k_\mathrm{B}T) - \sum_{i} k_\mathrm{B}T\ln(h\nu_i^{bcc}/k_\mathrm{B}T) \\ &= -\sum_{i\in\mathcal{S}} k_\mathrm{B}T\ln(h\nu_i^{fcc}/k_\mathrm{B}T) - \sum_{i\notin\mathcal{S}} k_\mathrm{B}T\ln(h\nu_i^{bcc}/k_\mathrm{B}T). \end{aligned}$$

which is a sum of the contributions of the stable modes of fcc and some of the stable modes of bcc (when the corresponding fcc mode is unstable). In the presence of anharmonicity, this simple expression does not hold exactly, but the essence of the argument remains.

A concern with this use of constrained free energy differences is that, in a system where there are multiple possible mechanically stable phases that can be used to reach the mechanically unstable phase, one would obtain a different free energy of the unstable phase using the different paths, because the stable modes from different mechanically stable phases would be copied over to replace the contributions of the unstable modes of the mechanically unstable phase.

A more practical concern with the use of constrained molecular dynamics techniques in this context is that it is not clear how to perform similar calculations for systems that are not high-symmetry pure elements, such as typical solid solution configurations. The problem goes beyond the large computational requirements. In general, the stable and unstable modes may not simply be separated by their wavelength, so even a small simulation cell may experience mechanical instability.

The method proposed in this paper offers a way to bypass the above problems.

Supplementary Note 3: Proof outline of the smoothness of the Helmholtz free energy

The proof that the Helmholtz free energy varies smoothly in response to changes in some external variable α, can be outlined as follows. The Helmholtz free energy associated with state σ in a system with potential $V(\mathbf{x}, \alpha)$ is given by $F_\sigma(\alpha) = -\beta^{-1} \ln \int_{\mathbf{x}\in\eta(\alpha)} \exp(-\beta V(\mathbf{x}, \alpha)) d\mathbf{x}$, where $\eta(\alpha)$ is the α-dependent neighborhood associated with some state σ. Differentiation of this expression with respect to α yields a sum of a $3N$ volume integral accounting for changes in $V(\mathbf{x}, \alpha)$ and a $3N - 1$ boundary integral accounting for changes in $\eta(\alpha)$. If $V(\mathbf{x}, \alpha)$ varies smoothly with α, so will the volume integral. The boundary of $\eta(\alpha)$ moves according to the position of the inflection points along paths of steepest decent. As shown above, this movement is also smooth, apart from exceptional points where both the second and third derivatives of the potential with respect to position along the path simultaneously vanish. This rare event would occur when two inflection points meet and then disappear as α varies, which corresponds to two neighborhoods η_σ and $\eta_{\sigma'}$ merging into one. So assuming that the states σ remain distinct as α varies rules this possibility out. We can even calculate higher order derivatives and observe that $F_\sigma(\alpha)$ essentially inherits the degree of smoothness of $V(\mathbf{x}, \alpha)$ in α, apart from exceptional cases where multiple derivatives of different order of the potential happen to simultaneously vanish.

Supplementary Note 4: Proof that $V(x^{\mathrm{r}}(\alpha), \alpha)$ is a continuously differentiable function of α.

We consider x to be one-dimensional (as this can be done without significant loss of generality by considering the potential along a path joining $\mathbf{x}^{\mathrm{u}}$ and $\mathbf{x}^{\mathrm{r}}$). We assume that the potential $V(x, \alpha)$ admits continuous mixed partial derivatives up to order 3 in x and order 1 in α. We also assume that $x^{\mathrm{u}}_\sigma \in \eta_\sigma$ for all values of α, so the association between a configuration σ and a neighborhood η_σ does not discontinuously change as α changes.

For $\alpha < \alpha_0$, the system is mechanically stable and the relaxed position $x^{\mathrm{r}}(\alpha)$ satisfies

$$V_x(x^{\mathrm{r}}(\alpha), \alpha) = 0,$$

where subscripts denote partial derivatives. The change in value of the potential at $x^{\mathrm{r}}(\alpha)$ as α changes is given by

$$\frac{d}{d\alpha} V(x^{\mathrm{r}}(\alpha), \alpha) = V_x(x^{\mathrm{r}}(\alpha), \alpha) x^{\mathrm{r}}_\alpha(\alpha) + V_\alpha(x^{\mathrm{r}}(\alpha), \alpha) = V_\alpha(x^{\mathrm{r}}(\alpha), \alpha)$$

since $V_x(x^{\mathrm{r}}(\alpha), \alpha) = 0$ for all $\alpha \le \alpha_0$ for a local minimum (this remains true even if $x^{\mathrm{r}}_\alpha(\alpha)$ diverges to infinity because $V_x(x^{\mathrm{r}}(\alpha), \alpha) = 0$ for all $\alpha \le \alpha_0$, so the product $V_x(x^{\mathrm{r}}(\alpha), \alpha) x^{\mathrm{r}}_\alpha(\alpha)$ remains zero at all $\alpha < \alpha_0$ and so is the limit as $\alpha \to \alpha_0$).

As shown in Supplementary Figure 1, for $\alpha < \alpha_0$, there are two other values of x satisfying $V_x(x, \alpha) = 0$: The local minimum at $x^{\mathrm{r}}_{\sigma'}$ (corresponding to the nearby mechanically stable phase) and a local maximum $x^{\mathrm{m}}(\alpha)$ necessarily located between the two local minima $x^{\mathrm{r}}(\alpha)$ and $x^{\mathrm{r}}_{\sigma'}$. There is also an inflection point $x^{\mathrm{i}}(\alpha)$ necessarily located between $x^{\mathrm{r}}(\alpha)$ and $x^{\mathrm{m}}(\alpha)$.

The force $V_x(x, \alpha)$ can be expanded as a quadratic form $q(x, \alpha)$ in x in the neighborhood of $x = x^{\mathrm{i}}(\alpha)$. For α close to α_0, the two zeros $x^{\mathrm{r}}(\alpha)$ and $x^{\mathrm{m}}(\alpha)$ of $V_x(x, \alpha)$ correspond to the two zeros of $q(x, \alpha)$. As $\alpha \to \alpha_0$ these two zeros must disappear and they do so by merging. Since the inflection point $x^{\mathrm{i}}(\alpha)$ lies between the zeros, as $\alpha \to \alpha_0$ all three points $x^{\mathrm{r}}(\alpha)$, $x^{\mathrm{i}}(\alpha)$ and $x^{\mathrm{m}}(\alpha)$ merge into a single point at $\alpha = \alpha_0$. Hence, at $\alpha = \alpha_0$ we simultaneously have

$$V_x(x^{\mathrm{i}}(\alpha_0), \alpha_0) = 0$$

and

$$V_{xx}(x^{\mathrm{i}}(\alpha_0), \alpha_0) = 0,$$

i.e., the local minimum and the inflection point are overlapping). Now, to compute $\frac{d}{d\alpha}V(x^{\mathrm{r}}(\alpha), \alpha)$ for $\alpha \geq \alpha_0$, we observe that

$$\frac{d}{d\alpha}V(x^{\mathrm{r}}(\alpha), \alpha) = \frac{d}{d\alpha}V(x^{\mathrm{i}}(\alpha), \alpha)$$

and that

$$\frac{d}{d\alpha}V(x^{\mathrm{i}}(\alpha), \alpha) = V_x(x^{\mathrm{i}}(\alpha), \alpha)x^{\mathrm{i}}_\alpha(\alpha) + V_\alpha(x^{\mathrm{i}}(\alpha), \alpha).$$

But at $\alpha = \alpha_0$ we also have $V_x(x^{\mathrm{i}}(\alpha_0), \alpha_0) = 0$ since the inflection point and minimum overlap, so

$$\frac{d}{d\alpha}V(x^{\mathrm{i}}(\alpha), \alpha) = V_\alpha(x^{\mathrm{i}}(\alpha), \alpha) = V_\alpha(x^{\mathrm{r}}(\alpha), \alpha)$$

at $\alpha = \alpha_0$, the exact same limiting value as $\alpha \to \alpha_0$ as for $\alpha < \alpha_0$. Hence the function $V(x^{\mathrm{r}}(\alpha), \alpha)$ is a continuously differentiable function of α at α_0.

(Rigorously, one also has to check that $x^{\mathrm{i}}_\alpha(\alpha)$ does not diverge as $\alpha \to \alpha_0$. This can be shown by taking the total differential with respect α of the definition of the inflection point $V_{xx}(x^{\mathrm{i}}(\alpha), \alpha) = 0$: $V_{xxx}(x^{\mathrm{i}}(\alpha), \alpha)x^{\mathrm{i}}_\alpha(\alpha) + V_{xx\alpha}(x^{\mathrm{i}}(\alpha), \alpha) = 0$, or $x^{\mathrm{i}}_\alpha(\alpha) = -V_{xx\alpha}(x^{\mathrm{i}}(\alpha), \alpha)/V_{xxx}(x^{\mathrm{i}}(\alpha), \alpha)$. We then observe that $V_{xxx}(x^{\mathrm{i}}(\alpha), \alpha)$ generally does not vanish as $\alpha \to \alpha_0$, apart from exceptional cases.)

We now show that the previous result cannot be extended to higher levels of smoothness — this is a fundamental and unavoidable feature for any method matching the local minimum when the system is mechanically stable. We have shown above that $x^{\mathrm{r}}(\alpha) \to x^{\mathrm{i}}(\alpha_0)$ as $\alpha \to \alpha_0$, so $x^{\mathrm{r}}(\alpha)$ is a continuous function of α. We now show that higher order derivatives are not continuous. For $\alpha \leq \alpha_0$, the local minimum $x^{\mathrm{r}}(\alpha)$ must satisfy $V_x(x^{\mathrm{r}}(\alpha), \alpha) = 0$ at all α and we can study how $x^{\mathrm{r}}(\alpha)$ changes with α by observing that a total derivative of that expression with respect to α must be zero as well: $V_{xx}(x^{\mathrm{r}}(\alpha), \alpha)x^{\mathrm{r}}_\alpha(\alpha) + V_{x\alpha}(x^{\mathrm{r}}(\alpha), \alpha) = 0$. Hence, $x^{\mathrm{r}}_\alpha(\alpha) = -V_{x\alpha}(x^{\mathrm{r}}(\alpha), \alpha)/V_{xx}(x^{\mathrm{r}}(\alpha), \alpha)$. But as $\alpha \to \alpha_0$, $x^{\mathrm{r}}(\alpha)$ converges to the inflection point so $V_{xx}(x^{\mathrm{r}}(\alpha), \alpha) \to 0$ as $\alpha \to \alpha_0$ and we have that $|x^{\mathrm{r}}_\alpha(\alpha)| \to \infty$ as $\alpha \to \alpha_0$. We conclude that $x^{\mathrm{r}}(\alpha)$ is only continuous at $\alpha = \alpha_0$ and not differentiable. To see the implication of this on $V(x^{\mathrm{r}}(\alpha), \alpha)$, we compute $\frac{d^2}{d\alpha^2}V(x^{\mathrm{r}}(\alpha), \alpha) = V_{xx}(x^{\mathrm{r}}(\alpha), \alpha)(x^{\mathrm{r}}_\alpha(\alpha))^2 + V_x(x^{\mathrm{r}}(\alpha), \alpha)x^{\mathrm{r}}_{\alpha\alpha}(\alpha) + 2V_{x\alpha}(x^{\mathrm{r}}(\alpha), \alpha)x^{\mathrm{r}}_\alpha(\alpha) + V_{\alpha\alpha}(x^{\mathrm{r}}(\alpha), \alpha)$. The first two terms may or may not diverge (depending on the specific shape of the potential) since there are the product of a function going to zero and one going to infinity. However, $V_{x\alpha}(x^{\mathrm{r}}(\alpha), \alpha)x^{\mathrm{r}}_\alpha(\alpha)$ clearly diverges because $V_{x\alpha}(x^{\mathrm{r}}(\alpha), \alpha)$ generally does not vanish (apart from exceptional cases that cannot be relied upon for a general theory), while $x^{\mathrm{r}}_\alpha(\alpha)$ was just shown to diverge as $\alpha \to \alpha_0$.

Supplementary Note 5: Additional details regarding Figure 4

1. In the harmonic and classical (i.e. high-temperature) approximations, the energy and entropy differences (arising from phonons) between fcc and bcc W are temperature-independent.

2. Quantum effects are not reported in Figure 4 because they are small at the temperatures of interest (where phase transitions occur) in this system.

3. Anharmonic effects are not considered but are likely to be small in our scheme, because we expand the potential in a region where it is closer to harmonic than it would be if we expanded around perfect fcc W.

4. Electronic contributions to the energy and entropy differences were calculated and are of the order of the size of the filled square (for temperature up to 3500K) and are thus not shown for clarity.

5. The same data is represented as a plot of free energy as a function of temperature in Supplementary Figure 2.

6. In the SGTE database, entries for Ir, Re and W, the differences between the free energies of two different phases of the same element are assumed linear in temperature. Hence, the energy and entropy contributions to the free energy difference are temperature-independent. This comment applies to Figure 3 as well.

Supplementary References

[1] Ozolins, V. First-principles calculations of free energies of unstable phases: The case of fcc W. *Phys. Rev. Lett.* **102**, 065702 (2009).

[2] Guillermet, A. F., Ozolins, V., Grimvall, G. & Krling, M. Phase stabilities in the Pt-W system: Thermodynamic and electronic-structure calculations. *Phys. Rev. B* **51**, 10364–10374 (1995).

[3] Saunders, N., Miodownik, A. & Dinsdale, A. Metastable lattice stabilities for the elements. *Calphad* **12**, 351–374 (1988).

[4] Dinsdale, A. T. SGTE data for pure elements. *Calphad* **15**, 317–425 (1991).

[5] Kaufman, L. & Bernstein, H. *Computer Calculation of Phase Diagrams* (Academic Press, New York, 1970).

[6] Sheppard, D., Xiao, P., Chemelewski, W., Johnson, D. D. & Henkelman, G. A generalized solid-state nudged elastic band method. *J. Chem. Phys.* **136**, 074103 (2012).

[7] van de Walle, A. & Ceder, G. The effect of lattice vibrations on substitutional alloy thermodynamics. *Rev. Mod. Phys.* **74**, 11–45 (2002).

[8] Grimvall, G., Magyari-Köpe, B., Ozolins, C. & Persson, K. A. Lattice instabilities in metallic elements. *Rev. Mod. Phys.* **84**, 945–986 (2012).

[9] Luo, W., Roundy, D., Cohen, M. L. & Morris, J. W. Ideal strength of bcc molybdenum and niobium. *Phys. Rev. B* **66**, 094110 (2002).

[10] Clatterbuck, D., Krenn, C. R., Cohen, M. L. & Morris, J. Phonon instabilities and the ideal strength of aluminum. *Phys. Rev. Lett.* **91**, 135501 (2003).
